# Anxiety Symptoms, COVID-19-Related Stress Reactions in the Italian General Population, and Validation of the Italian COVID Stress Scales (CSS-I)

**DOI:** 10.3390/jcm12175680

**Published:** 2023-08-31

**Authors:** Antonio Del Casale, Martina Nicole Modesti, Carlo Lai, Valeria Carola, Simone Mimun, Alba Bruzzese, Anna Maria Speranza, Dalainey H. Drakes, Gordon J. G. Asmundson, Giampaolo Nicolais

**Affiliations:** 1 Department of Dynamic and Clinical Psychology and Health Studies, Faculty of Medicine and Psychology, Sapienza University of Rome, 00185 Rome, Italyvaleria.carola@uniroma1.it (V.C.);; 2 Faculty of Medicine and Psychology, Sapienza University of Rome, Via di Grottarossa 1035, 00189 Rome, Italy; 3 School of Psychology, Faculty of Social Sciences, University of Ottawa, Ottawa, ON K1N 6N5, Canada; 4 Department of Psychology, Faculty of Arts, University of Regina, Regina, SK S4S 0A2, Canada

**Keywords:** COVID-19, Italian people, anxiety, depression, fear, compulsive behaviour, xenophobia, socioeconomic factors, psychometrics, reproducibility of results

## Abstract

*Background*. The COVID Stress Scales (CSS) assess COVID-related stress experienced in the past week related to danger and contamination fears, socioeconomic consequences, xenophobia, compulsive checking, and reassurance seeking, and traumatic stress symptoms. Our objective was to provide a translation into Italian, replication, and psychometric validation of the CSS in the general population. Moreover, we aimed to test the convergent and discriminant validity of the Italian CSS (CSS-I) with respect to anxiety, stress, and depressive symptoms in the general Italian population. *Method*. Adult participants (*n* = 935) over the age of 18 years were recruited from the general population in Italy. Psychological status was assessed using multiple validated measures, including the CSS, Depression, Anxiety and Stress Scales-21 (DASS-21), and the Prejudice Against Immigrants Scale (PAIS). *Results*. Our confirmatory factor analysis supported a 6-factor model, including danger fears (DAN), socioeconomic consequences (SEC), xenophobia (XEN), compulsive checking and reassurance seeking (CHE), contamination fears (CON), and traumatic stress symptoms (TSS). Strong reliability of the CSS-I (Cronbach’s α = 0.863–0.936) and convergent validity with the DASS-21 and PAI were established with positive correlations between total and scale scores across measures. *Conclusions*. The CSS-I is a valid and reliable instrument to measure COVID-19-related distress in the Italian population.

## 1. Introduction

On 31 December 2019, several cases of atypical pneumonia arose in Wuhan, China, caused by a novel coronavirus called Severe Acute Respiratory Syndrome CoronaVirus-2 (SARS-CoV-2) [1]. These cases spread worldwide, leading to the World Health Organization (WHO) declaration of a global pandemic on 11 March 2020. With the pandemic, critical consequences on mental health have emerged in the general population across nations [2,3,4,5,6]. Recent data support an increase in the prevalence of anxiety-, depression-, and stress-related disorders in the general population [7,8] and among health professionals [9,10]. Psychological distress related to extended periods of social isolation has been identified as a common stressor with a significant impact on elderly populations, individuals responding to greater financial burdens, women, and people responding to difficulties with accessing or managing communication technologies [8,11]. The pandemic has also led to additional complications that increased the burden on the general population via long periods of restriction to protect public health, social isolation via quarantine, increased rates of unemployment, and greater risk for and experience of intimate partner violence [2,12,13,14,15]. In Italy, the impact of the pandemic-related stress had consequences on the well-being of the Italian general population [16,17], including students [18], healthcare workers [19], and mothers [20], with a heavy burden on the Italian mental health system [21,22].

Some of the psychological difficulties triggered by the pandemic have specifically been associated with individuals who had experienced infection from SARS-CoV-2, including health anxiety, stigma-related concerns, amnesia, and traumatic memories of severe illness [5,23,24]. Nevertheless, the SARS-CoV-2 pandemic has contributed to an ongoing paradigm shift that acknowledges the role of interactions between biopsychosocial and contextual factors on the risk of experiencing consequential mental health difficulties or disorders. Infection with SARS-CoV-2 also presents the potential for the development of neurological and psychiatric sequelae via direct effects on the central nervous system (CNS) (e.g., rare direct infection of nerve ends) as well as indirect effects of medical therapy or abnormal immune response (e.g., immune system malfunction and vascular system dysregulation) [2,25].

Throughout the SARS-CoV-2 pandemic, many people showed anxiety-related distress, expressed through fears of becoming infected, possibly through contaminated objects or surfaces, fear of foreigners who might be infected, and fear of the socioeconomic consequences of the pandemic. Psychological distress has also been associated with compulsive checking and reassurance seeking about pandemic-related threats and traumatic stress symptoms [2,3,4,6,26,27].

Anxiety and depressive symptoms, or the lack thereof, are essential drivers of behaviour during pandemics [26,28]. For example, people showing lower levels of anxiety with respect to viral infection outbreaks are less likely to engage in hygiene behaviours, adhere to physical distancing measures, and are more apprehensive about being vaccinated [26]. It is plausible that some individuals living with depression experience greater impairment from their disorder, as opposed to presenting concerns associated with the pandemic, as observed in Italian patients [28]. Furthermore, anxiety has been associated with the risk of socially disruptive behaviours, including panic buying and unnecessary access to healthcare facilities [7,26,29,30].

The COVID Stress Scales (CSS) were developed to measure fear, worry, and stress symptoms to better understand and assess COVID-19-related distress. These validated scales were intentionally designed to be readily adapted to future pandemics [31]. Other groups have shown that SARS-CoV-2-related stress is (1) predictive of future career anxiety in college students [32] and (2) associated with greater intimate partner violence in economically disadvantaged people [33], and (3) that traumatic stress symptoms and xenophobia, in particular, are associated with increased odds of suicidal ideation in essential workers, especially those who self-identify as Black, Indigenous, and people of colour [34]. The SARS-CoV-2 pandemic has sparked a surge in xenophobia, fuelled by the association of the virus with certain countries or cultures. Misinformation, scapegoating, and travel restrictions have worsened discriminatory attitudes. Xenophobia specifically focuses on fear of or aversion to foreigners or those perceived as different, whereas prejudice is a more general term for holding negative attitudes and beliefs about any group of people based on various factors. Xenophobia can be considered a subset of prejudice that is specifically rooted in fear of the foreign or unfamiliar. Addressing and combating prejudice and xenophobia is crucial for fostering inclusivity, unity, and global solidarity in a post-pandemic world.

Pandemics are dynamic rather than static; therefore, stress-related symptoms can change over time. As such, it has been demonstrated that SARS-CoV-2-related stress symptoms have also changed across various pandemic waves, with higher levels reported during the early phases of the pandemic, when the perception of threat was greatest in the general population, and lower during the later phases, when vaccines were deployed and the threat perception was reduced [35]. Despite the SARS-CoV-2 pandemic transitioning to an endemic phase, individuals continue to acquire variants of the SARS-CoV-2 infection (XBB.1.16, 1.9.1, 2.3), and specific populations within the general population continue to be at higher risk for infection than others (e.g., immunocompromised populations, elderly) [36]. Even as we transition to post-pandemic life, it is crucial to monitor SARS-CoV-2-related stress levels. Ongoing stressors, including financial challenges and health concerns, can persist despite the declared end of the pandemic, and evaluating acute stress levels helps us understand their impact. Prolonged stress can have enduring effects on mental health, making it essential to assess SARS-CoV-2-related stress levels to measure recovery progress and identify areas that require support. Moreover, it is likely that stress will be a core facet of future pandemics. In the present study, we examined the factor structure, reliability as internal consistency, and convergent and discriminant validity of the Italian translation of the CSS (CSS-I) in the general Italian population approximately 2.5 years following the declaration of the pandemic.

## 2. Materials and Methods

### 2.1. Participants and Procedure

Two independent translators (MNM, ADC) performed forward translation (English to Italian) and back translation (Italian to English) of the CSS. Four researchers (MNM, ADC, DHD, and GJGA) compared the original English CSS to the back-translated CSS-I. Minor wording discrepancies were identified and resolved after re-consultation with the translators. During initial pilot testing, the Italian terms were found to be satisfactory. We report the CSS-I in Supplementary Table S1.

We recruited participants among the general Italian population from 13 May 2022 to 29 September 2022. The questionnaire was aimed only at the adult population, so the sole exclusion criterion was being underage. The study received approval by the Ethical Committee of Sapienza University, Department of Dynamic and Clinical Psychology and Health Studies (Protocol number 0000596). Data were collected through a web panel based on a Google module. Informed consent was provided by all participants who agreed to voluntarily complete the administered measures, which were completed in approximately 10 min.

### 2.2. Measures

We collected the following sociodemographic and clinical variables of participants: gender, age, nationality, parent status, years of education, work status (student/employed/unemployed/retired), and history of mental disorders.

COVID Stress Scales [31]: The CSS consists of 36 items distributed over 6 scales, including danger-related fears (DAN), socioeconomic consequences (SEC), xenophobia (XEN), contamination fears (CON), traumatic stress symptoms (TSS), and compulsive checking and reassurance seeking (CHE). The 6 scales assess various aspects of COVID-19-related distress over the past seven days. According to Taylor et al. [31], DAN and CON subscales converged onto the same factor, which suggested a final 5-factor solution (DANCON, SEC, XEN, TSS, and CHE). These distinct scales are highly correlated (Taylor et al. [31]), and both total and single scale scores can be used for assessment, in which higher scores indicate more significant SARS-CoV-2-related distress. Consistent evidence supports the CSS validity and reliability as well as its stability across cultures [26,31,37,38,39,40].

*Depression Anxiety Stress Scales-21* (DASS-21) [41]: These scales measure general distress and three additional orthogonal dimensions (e.g., anxiety, depression, and stress). These scales have good internal consistency and temporal stability, good convergent and divergent validity, and good criterion-oriented validity. The validated Italian version of the DASS-21 was utilized [42].

*Prejudice Against Immigrants Scale* (PAIS) [43]: The PAIS is a valid and reliable instrument to measure prejudice against immigrants. The validated Italian version was administered with a related two-factor solution, in which the first factor is “classical prejudice against immigrants” and the second is “conditional prejudice against immigrants” (i.e., subtle and modern prejudices) [44].

### 2.3. Statistical Analyses

We used the software IBM SPSS Statistics 27.0.1 (IBM Corp, Armonk, NY, USA) for descriptive analyses and the JASP software v. 0.16.3 [45] to conduct confirmatory factor analysis (CFA). Consequently, following an evaluation of the data’s distribution by examining the skewness and kurtosis values of the residuals obtained from the Z values of the study scales, we proceeded to conduct parametric tests. We investigated differences in continuous variables between men and women through analysis of variance (one-way ANOVA). We analysed differences in categorical variables through the chi-square test. Considering the objectives of our study (i.e., replication and validation of the CSS), our analyses were informed by the related constructs of the original manuscript published by Taylor et al. [31]. We conducted a CFA to show the goodness of fit of both the theoretical six-factor model and the empirical five-factor model from the analyses reported by Taylor et al. [31]. The goodness of fit was determined based on the root-mean-square error of approximation (RMSEA), standardized square residual (SRMR), and comparative fit index (CFI). These goodness of fit indices were compared with empirically informed cutoff values that reduce the likelihood for effort where CFI ≥ 0.90 signifies a good fit and an excellent fit is represented by RMSEA ≤ 0.06, SRMR ≤ 0.08, and CFI ≥ 0.95 [46,47].

We analysed the correlations between the CSS subscales and the other assessment instruments (i.e., DASS-21, PAIS) by the Pearson correlation test (2-sided *p*). Furthermore, we conducted a linear regression analysis to investigate how sex, age, and education related to the CSS-I. We used the CSS-I total score as the dependent variable and sex, age, and years of education as independent variables.

## 3. Results

We recruited 935 participants from the general Italian population (*M_Age_* = 39.32 years; *SD* = 14.37; age range: 18–86), with most participants having self-identified as females (55.3%). Age did not differ statistically between males and females (*F* = 0.929; *p* = 0.395), and gender composition did not differ significantly compared with the general adult Italian population (*χ*^2^ = 2.65; *p* = 0.103) on 1 January 2022, as reported by the National Institute of Statistics (ISTAT) [48]. Most participants self-identified with Italian nationality (97.6%), and the majority reported a relationship status of being single (36.6%) or coupled/married (57.5%). Participants were most often employed (73.2%) or students (18.1%) residing in Northern Italy (18.5%), Central Italy (51.9%), and Southern Italy/islands (28.8%). Among the entire sample, 19.3% of participants reported being affected by a generic history of a mental disorder, i.e., an anxiety disorder (9.2%), major depressive disorder (5.6%), bipolar disorder (0.5%), or other mental disorders (3.9%). We report the main sociodemographic aspects of our sample in Table 1.

The residuals of the CSS-I, PAIS, and DASS-21 factors exhibited a skewness value of 0.556 (SE = 0.086) and a kurtosis value of −0.257 (SE = 0.171), suggesting an approximately normal distribution, given the substantial sample size.

We tested the model fit of both the original 6-factor model and the 5-factor model of CSS reported by Taylor et al. [31]. In our Italian sample, the CSS 6-factor model indicated a good model fit, with RMSEA = 0.077 (90% CI: 0.074–0.079), SRMR = 0.062, GFI = 0.801, and CFI = 0.874. The CSS 5-factor model also indicated a good model fit, with RMSEA = 0.090 (90% CI: 0.088–0.092), SRMR = 0.066, GFI = 0.745, and CFI = 0.824. The CSS 6-factor and 5-factor models performed well in our Italian sample; however, the 6-factor model had a slightly better fit than the 5-factor model.

The CSS-I showed the Cronbach’s α = 0.947 (36 items), the PAISS α was 0.931 (16 items), and the DASS-21 scale α was 0.960 (21 items). Each CSS-I subscale was confirmed in our CFA, including danger fears (items 1–6; Cronbach’s α = 0.865), fears about economic consequences (items 7–12; Cronbach’s α = 0.916), xenophobia (items 13–18; Cronbach’s α = 0.936), contamination fears (items 19–24; Cronbach’s α = 0.912), COVID-19 traumatic stress symptoms (items 25–30; Cronbach’s α = 0.914), and compulsive checking and reassurance seeking (items 31–36; Cronbach’s α = 0.863).

**Figure 1:**
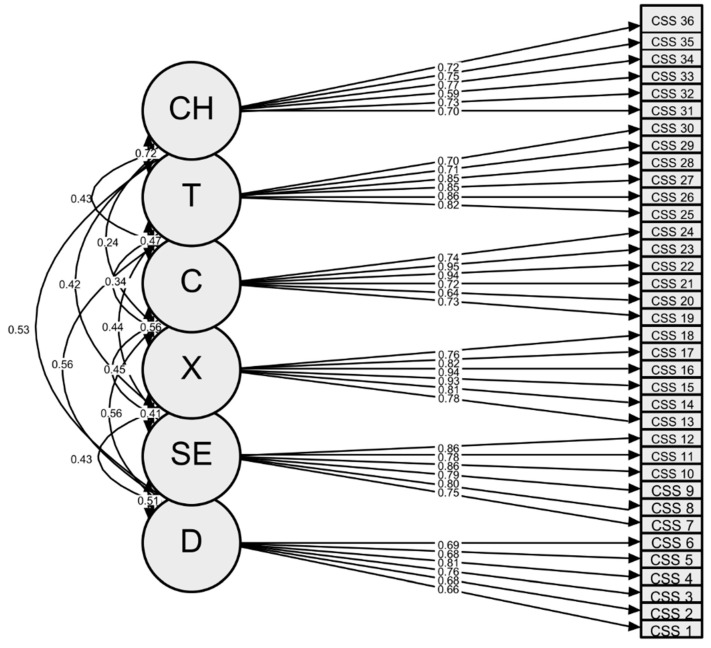
CFA plot. Legend. C: contamination fears; CH: compulsive checking and reassurance seeking; D: danger fears; SE: fears about economic consequences; T: traumatic stress symptoms; X: xenophobia.

Items within each factor showed a high linear correlation, and Cronbach’s alpha value was compatible with good internal consistency. We summarised the CSS-I 6-factor structure in Table 2a–c and the CFA plot in Figure 1.

The CSS-I subscales were highly correlated with the other assessment instrument subscales regarding general distress, anxiety, and depressive symptoms assessed with the DASS-21, and classical and conditional prejudices assessed with the PAIS. We summarised these correlations in Table 3.

The multiple regression analysis (Table 4) conducted to investigate how sex, age, and education related to the CSS-I showed that the female sex (*β* = −0.122; *p* < 0.001), older age (*β* = 0.174; *p* < 0.001), and a lower level of education (*β* = −0.09; *p* = 0.008) were significantly related to higher CSS-I. The overall regression was statistically significant (*R^2^* = 0.059, F = 17.829, *p* < 0.001), although explained only about 6% of the variance in CSS-I.

## 4. Discussion

Following the development and validation of the CSS by Taylor et al. [31], as well as various other language translations (e.g., Swedish, Chinese, Serbian, Hungarian, and Spanish), we aimed to translate and validate the CSS-I in a population-representative sample from Italy. We replicated the results of Taylor et al. [31] and other validations of the CSS in other languages or cultures [37,39,40,49,50,51]. Our results support the applicability of the 6-factor model of the CSS using the CSS-I. Analyses of the total and scale scores support that the CSS-I demonstrates strong psychometric properties and serves as an excellent transdiagnostic tool for assessing emotional distress associated with contagious viral outbreaks. Significant convergent validity and correlations between the CSS-I and anxiety, depression, and stress assessment as measured by DASS-21 were identified. The CSS-I notably correlated with anxiety, depression, and stress-related symptoms. The CSS-I was also compared with the PAIS to further measure convergent validity with xenophobia, demonstrating significant correlations with both classical and conditional prejudice against immigrants, notably for the XEN factor. By combining these two instruments, we aimed to analyse and differentiate between general xenophobia and prejudice towards immigrants, resulting in a more comprehensive understanding of negative attitudes towards immigrant populations. This approach strengthens the validity and reliability of our findings, offering a deeper exploration of the complex nature of these attitudes. Furthermore, the significant correlations we found between both classical and conditional prejudice against immigrants and the CSS XEN factor confirm that xenophobia can be considered a subset of prejudice that is specifically rooted in a fear of the foreign or unfamiliar and that these subdimensions coexist and describe the distinctiveness of aspects of fear towards foreigners and towards infectious pathologies, i.e., in this case, SARS-CoV-2.

Taylor et al. [31] demonstrated that age negatively correlated with the total CSS score in samples from the United States and Canada, while women and people with fewer years of education had higher CSS total scores. In our Italian sample, we found a minimal effect of sex, age, or education on total CSS-I scores. However, older age predicted a higher total CSS-I that parallels the findings of the Swedish CSS validation data [37]. Furthermore, in our Italian sample, a lower level of education and female sex predicted a higher total CSS-I, as observed in North America and Sweden [31,37]. These results suggest that younger age and higher levels of education may serve as protective factors against COVID-related stress.

The CSS was based on evidence and clinical experiences with previous pandemics and outbreaks [31]. The CSS, and its various translations, which now include the CSS-I, is a valuable assessment instrument in the context of the current COVID-19 pandemic, regarding all known and potential SARS-CoV-2 variants, and in the context of potential other outbreaks. Our findings are applicable to the Italian population, and our tool can facilitate a comprehensive understanding of the psychological impact of the pandemic, guiding interventions and promoting resilience. Furthermore, this scale contributes to comprehending nuanced emotional reactions triggered by the pandemic, enabling targeted interventions and appropriate support systems. Additionally, it proves to be a valuable instrument for researchers to monitor the changing nature of stressors, facilitating the development of flexible strategies for mental health promotion. Policy makers can utilize data from the scale to formulate evidence-based policies addressing the mental health consequences of the pandemic.

It is essential to remember that the course of the pandemic followed many changes throughout the years, as there have been various waves of infection globally over the more than 3-year course of SARS-CoV-2. In this regard, as the original CSS had been developed following the first waves, the pandemic stress may have moderated throughout time and during data collection for the Italian CSS, despite maintaining the same trends. For this reason, no cross-national comparisons at various time points during the pandemic were made in the current study.

Our study has some limitations that warrant consideration and might help guide future research. We did not investigate test-retest reliability or predictive validity, which could be examined with longitudinal research in the future. Second, future research could develop and evaluate objective measures of SARS-CoV-2-related stress, including physiological stress signs, which could be incorporated with the CCS in laboratory or ecologically based experiments. However, the main aim of this study was to validate the CSS in the Italian language, and these limitations do not regard this point. The recruited sample, indeed, is representative of the Italian general population, also considering that the reported prevalence of mental disorders in our sample is similar to that of the general population [48,52]. In addition, the Italian CSS is a valid translation of the CSS for use in the Italian language to assess for SARS-CoV-2-related stress in the ongoing pandemic and future pandemics. Last, as an important strength point, the 6-factor model of CSS has cross-cultural applications to the Italian population, signifying that CSS has an impact beyond the original North American samples and extends to those residing in Italy as well. The reliability of the CSS across multiple cultures has been extensively documented, and our findings regarding reliability are consistent with those obtained in validation studies conducted in different countries [53].

## 5. Conclusions

Development and validation of the Italian translation of the CSS in a population-representative sample were established. Our results support the validity of the CSS-I and its utility in assessing xenophobia, fears about economic consequences, traumatic stress symptoms, contamination fears, danger fears, and compulsive checking and reassurance seeking related to current and future pandemics.

## Figures and Tables

**Table 1:** Sociodemographic characteristics of the sample.

	N (SD)
Gender (f/m/ns)	517/412/6
Mean age (years)	39.32 (14.37)
Nationality (ITA/Other EU countries/Extra-EU countries/ns)	913/7/12/8
Parent status (yes/no)	351/576
Mean education (years)	16.62 (3.6)
Work status (student/employed/unemployed/retired/ns)	169/684/44/37

Legend. EU: European Union; ITA: Italy; Ns: not specified; SD: standard deviation.

**Table 2:** (**a**) CSS-I 6-factor model fit; (**b**) Other fit measures; (**c**) CSS-I CFA: factor covariances.

(**a**)										
**Model**				**Χ²**			**df**	***p***		
Baseline model				24,712.957			630			
Factor model				3624.845			579	<0.001		
**Fit indices**										
**Index**								**Value**		
Comparative Fit Index (CFI)								0.874		
Tucker–Lewis Index (TLI)								0.862		
Bentler–Bonett Non-normed Fit Index (NNFI)								0.862		
Bentler–Bonett Normed Fit Index (NFI)								0.853		
Parsimony Normed Fit Index (PNFI)								0.784		
Bollen’s Relative Fit Index (RFI)								0.840		
Bollen’s Incremental Fit Index (IFI)								0.874		
Relative Noncentrality Index (RNI)								0.874		
**Information criteria**										
								**Value**		
Log-likelihood								−36,035.550		
Number of free parameters								87.000		
Akaike (AIC)								72,245.099		
Bayesian (BIC)								72,662.131		
Sample-size-adjusted Bayesian (SSABIC)								72,385.835		
(**b**)										
**Metric**								**Value**		
Root-mean-square error of approximation (RMSEA)								0.077		
RMSEA 90% CI lower bound								0.074		
RMSEA 90% CI upper bound								0.079		
RMSEA *p*-value								<0.001		
Standardized root-mean-square residual (SRMR)								0.062		
Hoelter’s critical N (α = 0.05)								157.528		
Hoelter’s critical N (α = 0.01)								163.681		
Goodness of fit index (GFI)								0.801		
McDonald’s fit index (MFI)								0.181		
Expected cross-validation index (ECVI)								4.259		
(**c**)										
**Factor Covariances**										
								**95% Confidence Interval**		
			**Estimate**		**Std. Error**	**z-Value**		***p***	**Lower**	**Upper**
D	↔	SE	0.508		0.029	17.655		<0.001	0.452	0.565
D	↔	X	0.435		0.030	14.268		<0.001	0.375	0.494
D	↔	C	0.558		0.026	21.151		<0.001	0.507	0.610
D	↔	T	0.561		0.027	20.824		<0.001	0.508	0.614
D	↔	CH	0.532		0.029	18.160		<0.001	0.474	0.589
SE	↔	X	0.406		0.030	13.479		<0.001	0.347	0.465
SE	↔	C	0.446		0.029	15.364		<0.001	0.389	0.503
SE	↔	T	0.438		0.030	14.655		<0.001	0.379	0.496
SE	↔	CH	0.419		0.032	13.252		<0.001	0.357	0.481
X	↔	C	0.555		0.025	22.396		<0.001	0.507	0.604
X	↔	T	0.339		0.032	10.646		<0.001	0.277	0.401
X	↔	CH	0.237		0.035	6.764		<0.001	0.168	0.305
C	↔	T	0.469		0.028	16.564		<0.001	0.414	0.525
C	↔	CH	0.433		0.031	14.124		<0.001	0.373	0.493
T	↔	CH	0.723		0.020	35.819		<0.001	0.684	0.763

Legend. C: contamination fears; CH: compulsive checking and reassurance seeking; D: danger fears; SE: fears about economic consequences; T: traumatic stress symptoms; X: xenophobia.

**Table 3:** Pearson correlations between study variables.

		CSS D	CSS SE	CSS X	CSS C	CSS T	CSS CH	CSS Total	PAIS F2 Classic Prejudice	PAIS F2 Conditional Prejudice	DASS F1 Depression	DASS F2 Anxiety	DASS F3 Stress	DASS Total Score
CSS D	Pearson Correlation	1	0.456 **	0.420 **	0.598 **	0.526 **	0.466 **	0.796 **	0.186 **	0.163 **	0.278 **	0.369 **	0.308 **	0.336 **
	*p*		<0.001	<0.001	<0.001	<0.001	<0.001	<0.001	<0.001	<0.001	<0.001	<0.001	<0.001	<0.001
	N	926	922	913	916	922	919	892	911	908	914	913	913	896
CSS SE	Pearson Correlation	0.456 **	1	0.398 **	0.432 **	0.392 **	0.376 **	0.670 **	0.244 **	0.196 **	0.242 **	0.347 **	0.225 **	0.288 **
	*p*	<0.001		<0.001	<0.001	<0.001	<0.001	<0.001	<0.001	<0.001	<0.001	<0.001	<0.001	<0.001
	N	922	930	918	921	926	921	892	914	911	918	916	916	898
CSS X	Pearson Correlation	0.420 **	0.398 **	1	0.570 **	0.319 **	0.223 **	0.661 **	0.551 **	0.443 **	0.094 **	0.135 **	0.072 *	0.106 **
	*p*	<0.001	<0.001		<0.001	<0.001	<0.001	<0.001	<0.001	<0.001	0.005	<0.001	0.029	0.001
	N	913	918	921	913	917	912	892	907	904	909	908	907	890
CSS C	Pearson Correlation	0.598 **	0.432 **	0.570 **	1	0.483 **	0.426 **	0.797 **	0.238 **	0.215 **	0.245 **	0.348 **	0.264 **	0.303 **
	*p*	<0.001	<0.001	<0.001		<0.001	<0.001	<0.001	<0.001	<0.001	<0.001	<0.001	<0.001	<0.001
	N	916	921	913	924	920	915	892	908	905	913	911	910	894
CSS T	Pearson Correlation	0.526 **	0.392 **	0.319 **	0.483 **	1	0.645 **	0.759 **	0.134 **	0.100 **	0.391 **	0.526 **	0.418 **	0.469 **
	*p*	<0.001	<0.001	<0.001	<0.001		<0.001	<0.001	<0.001	0.002	<0.001	<0.001	<0.001	<0.001
	N	922	926	917	920	931	923	892	915	911	919	917	917	899
CSS CH	Pearson Correlation	0.466 **	0.376 **	0.223 **	0.426 **	0.645 **	1	0.714 **	0.131 **	0.108 **	0.312 **	0.441 **	0.357 **	0.398 **
	*p*	<0.001	<0.001	<0.001	<0.001	<0.001		<0.001	<0.001	0.001	<0.001	<0.001	<0.001	<0.001
	N	919	921	912	915	923	926	892	911	907	914	912	913	895
CSS total score	Pearson Correlation	0.796 **	0.670 **	0.661 **	0.797 **	0.759 **	0.714 **	1	0.314 **	0.257 **	0.352 **	0.485 **	0.370 **	0.428 **
	*p*	<0.001	<0.001	<0.001	<0.001	<0.001	<0.001		<0.001	<0.001	<0.001	<0.001	<0.001	<0.001
	N	892	892	892	892	892	892	892	879	877	881	881	879	865
PAIS F1 classic prejudice	Pearson Correlation	0.186 **	0.244 **	0.551 **	0.238 **	0.134 **	0.131 **	0.314 **	1	0.713 **	0.061	0.086 **	0.052	0.073^*^
	*p*	<0.001	<0.001	<0.001	<0.001	<0.001	<0.001	<0.001		<0.001	0.065	0.009	0.115	0.030
	N	911	914	907	908	915	911	879	919	906	908	905	907	889
PAIS F2 conditional prejudice	Pearson Correlation	0.163 **	0.196 **	0.443 **	0.215 **	0.100 **	0.108 **	0.257 **	0.713 **	1	0.100 **	0.081 *	0.101 **	0.104 **
	*p*	<0.001	<0.001	<0.001	<0.001	0.002	0.001	<0.001	<0.001		0.003	0.016	0.002	0.002
	N	908	911	904	905	911	907	877	906	915	904	901	904	886
DASS F1 Depression	Pearson Correlation	0.278 **	0.242 **	0.094 **	0.245 **	0.391 **	0.312 **	0.352 **	0.061	0.100 **	1	0.728 **	0.826 **	0.931 **
	*p*	<0.001	<0.001	0.005	<0.001	<0.001	<0.001	<0.001	0.065	0.003		<0.001	<0.001	<0.001
	N	914	918	909	913	919	914	881	908	904	923	911	914	903
DASS F2 Anxiety	Pearson Correlation	0.369 **	0.347 **	0.135 **	0.348 **	0.526 **	0.441 **	0.485 **	0.086 **	0.081 *	0.728 **	1	0.751 **	0.882 **
	*p*	<0.001	<0.001	<0.001	<0.001	<0.001	<0.001	<0.001	0.009	0.016	<0.001		<0.001	<0.001
	N	913	916	908	911	917	912	881	905	901	911	921	910	903
DASS F3 Stress	Pearson Correlation	0.308 **	0.225 **	0.072 *	0.264 **	0.418 **	0.357 **	0.370 **	0.052	0.101 **	0.826 **	0.751 **	1	0.941 **
	*p*	<0.001	<0.001	0.029	<0.001	<0.001	<0.001	<0.001	0.115	0.002	<0.001	<0.001		<0.001
	N	913	916	907	910	917	913	879	907	904	914	910	921	903
DASS total score	Pearson Correlation	0.336 **	0.288 **	0.106 **	0.303 **	0.469 **	0.398 **	0.428 **	0.073 *	0.104 **	0.931 **	0.882 **	0.941 **	1
	*p*	<0.001	<0.001	0.001	<0.001	<0.001	<0.001	<0.001	0.030	0.002	<0.001	<0.001	<0.001	
	N	896	898	890	894	899	895	865	889	886	903	903	903	903

Legend. C: contamination fears; CH: compulsive checking and reassurance seeking; CSS: COVID Stress Scales; D: danger fears; DASS: Depression Anxiety Stress Scales; F1: factor 1; F2: factor 2; F3: factor 3; PAIS: Prejudice Against Immigrants Scale; SE: fears about economic consequences; T: traumatic stress symptoms; X: xenophobia; ** Correlation is significant at the 0.01 level (2-tailed); * Correlation is significant at the 0.05 level (2-tailed).

**Table 4:** Multiple linear regression analysis, COVID Stress Scales (CSS-I) total scores regressed on sex, age, and years of education.

	β	B	SE	*p*	95% CI Lower	95% CI Upper
Age	0.174	0.275	0.053	<0.001	0.171	0.379
Gender (male)	−0.122	−5.385	1.489	<0.001	−8.307	−2.463
Years of education	−0.09	−0.561	0.212	0.008	−0.977	−0.144
Constant		32.314	4.314	<0.001	23.847	40.781

## Data Availability

Available upon request for a justified reason by contacting the corresponding author.

## Supplementary Materials

The following supporting information can be downloaded at: https://www.mdpi.com/article/10.3390/jcm12175680/s1, Table S1: Italian COVID stress scales (CSS-I).
