# Supplementary material for: Anxiety Symptoms, COVID-19-Related Stress Reactions in the Italian General Population, and Validation of the Italian COVID Stress Scales (CSS-I)
Source: J Clin Med. 2023 Aug 31;12(17):5680. doi: 10.3390/jcm12175680 (PMC10488780; doi:10.3390/jcm12175680)
Supplement: Supplementary file 1 [file jcm-12-05680-s001.zip › jcm-12-05680-supplementary.pdf]

Table S1: Italian COVID stress scales (CSS-I)

Le seguenti domande fanno riferimento a timori che potresti aver sperimentato negli ultimi 7 giorni. Nelle frasi seguenti, quando si parla di virus, si fa riferimento al Covid-19.

		Per nulla	Poco	Abbastanza	Molto	Moltissimo
1	Ho paura di contrarre il virus	0	1	2	3	4
2	Ho paura di non riuscire a proteggere i miei cari dal virus	0	1	2	3	4
3	Ho paura che il sistema sanitario non riesca a proteggere i miei cari	0	1	2	3	4
4	Ho paura che il sistema sanitario non mi possa proteggere dal virus	0	1	2	3	4
5	Ho paura che le misure igieniche consigliate (es. lavarsi le mani) non siano sufficienti per proteggersi dal virus	0	1	2	3	4
6	Ho paura che il distanziamento sociale non sia sufficiente a proteggermi dal virus	0	1	2	3	4
7	Ho paura che termini il cibo nei supermercati	0	1	2	3	4
8	Ho paura che chiudano i supermercati	0	1	2	3	4
9	Ho paura che nei supermercati finiscano i disinfettanti o i prodotti per pulire	0	1	2	3	4
10	Ho paura che finiscano le medicine per curare il raffreddore o l'influenza	0	1	2	3	4
11	Ho paura che termini l'acqua nei supermercati	0	1	2	3	4
12	Ho paura che terminino i farmaci nelle farmacie	0	1	2	3	4
13	Ho paura che persone straniere diffondano il virus nel mio paese	0	1	2	3	4
14	Se vado in un ristorante etnico, ho paura di prendere il virus	0	1	2	3	4
15	Ho paura di venire a contatto con una persona straniera, perché potrebbe avere il virus	0	1	2	3	4
16	Se facessi la conoscenza di una persona straniera, avrei il timore che possa avere il virus	0	1	2	3	4
17	Se fossi in ascensore con un gruppo di stranieri, avrei il timore che abbiano il virus	0	1	2	3	4
18	Ho il timore che gli stranieri trasmettano di più il virus perché hanno un'igiene più scarsa	0	1	2	3	4
19	Ho paura che se tocco qualcosa in uno spazio pubblico (es. maniglia sull'autobus) prendo il virus	0	1	2	3	4
20	Ho paura che se qualcuno tossisca o starnutisca vicino a me, mi possa infettare	0	1	2	3	4
21	Ho paura che le persone intorno a me possano infettarmi	0	1	2	3	4
22	Ho paura di infettarmi se prendo il resto quando pago in contanti	0	1	2	3	4

23	Ho paura di potermi infettare quando prelevo contante o uso contante	0	1	2	3	4
24	Ho paura che la mia posta possa essere contaminata dai postini	0	1	2	3	4

Per favore leggi le frasi attentamente e indica quanto spesso ti è capitato negli ultimi 7 giorni di sperimentare il problema descritto.

25	Ho avuto problemi a concentrarmi perché non riuscivo a smettere di pensare al virus	0	1	2	3	4
26	Mi è capitato che mi venissero in mente, senza che lo volessi, immagini brutte riguardo il virus	0	1	2	3	4
27	Ho avuto problemi a dormire per le preoccupazioni legate al virus	0	1	2	3	4
28	Mi è venuto da pensare al virus anche quando non lo volevo	0	1	2	3	4
29	Notizie sul virus mi fanno venire reazioni fisiche, come sudorazione e palpitazioni	0	1	2	3	4
30	Ho fatto incubi sul virus	0	1	2	3	4

Le domande seguenti si riferiscono ad alcuni comportamenti. Quante volte, negli ultimi 7 giorni, ti è capitato di fare una di queste cose perché eri preoccupato per il Covid-19?

31	Ho cercato su internet cure per il Covid-19	0	1	2	3	4
32	Ho chiesto a medici e farmacisti informazioni sul Covid-19	0	1	2	3	4
33	Ho guardato video su Youtube riguardo il Covid-19	0	1	2	3	4
34	Ho controllato se avessi segni/sintomi del Covid-19 (es. misurato la temperatura corporea)	0	1	2	3	4
35	Ho cercato rassicurazioni sul virus dagli amici e dalla famiglia	0	1	2	3	4
36	Ho controllato sui social media nuovi post sul virus	0	1	2	3	4
